# Germline *BRCA1* and *BRCA2* mutations and the risk of bladder or kidney cancer in Poland

**DOI:** 10.1186/s13053-022-00220-6

**Published:** 2022-04-08

**Authors:** Elżbieta Złowocka-Perłowska, Aleksandra Tołoczko-Grabarek, Steven A. Narod, Jan Lubiński

**Affiliations:** 1 Department of Genetics and Pathology, International Hereditary Cancer Center, Pomeranian Medical University, Szczecin, Poland; 2 Women’s College Research Institute, Women’s College Hospital, University of Toronto, Toronto, Canada; 3 Dalla Lana School of Public Health, University of Toronto, Toronto, Canada

**Keywords:** Mutation: 5328 insC, C61G, 4153 delA, C5972T; gene: *BRCA1*, *BRCA2*

## Abstract

**Introduction:**

The role of the *BRCA1* and *BRCA2* genes in bladder and renal tumorigenesis is unclear. Our goal was to determine the prevalence of specific founder mutations genes *BRCA1* (5328 insC, C61G and 4153 delA) and *BRCA2* (C5972T) mutations in bladder and kidney cancer patients from Poland.

**Materials and methods:**

We genotyped 1028 patients with bladder cancer and 688 cases with kidney cancer and two control groups*.*

**Results:**

A *BRCA1* mutation (all variants combined) was detected in peripheral blood leukocytes in 5 out of 1028 (0.5%) bladder cases and in 17 of 4000 controls (0.4%) (odds ratio [OR], (OR = 1.1; 95% CI 0.42–3.11; *p* = 1.0). Among 688 unselected kidney cancer cases a *BRCA1* mutations was reported in three patients (0.4%) (OR = 1.0; 95% CI 0.29–3.51; *p* = 1.0). The mutation C5972T in *BRCA2* was observed in 54 bladder cancer patients (5.2%) and in 159 of 2791 healthy controls (5.7%) (OR = 0.9; 95% CI 0.66–1.26; *p* = 0.6). Fifty kidney cancer cases carried a *BRCA2* mutation (7.3%) (OR = 1.3; 95% CI 0.93–1.80; *p* = 0.1).

**Conclusion:**

In conclusion, we found no difference in the prevalence of *BRCA1* and *BRCA2* founder mutations between cases and healthy controls. The mutations *BRCA1* and *BRCA2* seem not to play a role in bladder and kidney cancer development in Polish patients.

## Introduction

Bladder cancer is the second most common malignant neoplasm of the genitourinary system in men. Bladder malignancies are responsible for approximately 5% of deaths in men and 2% in women. Currently, kidney cancer accounts for approximately 3% of all cancers diagnosed in adults. Malignant neoplasms account for approximately 3% of male deaths and 2% of female cancers [1]. So far, the molecular basis for the development of a small percentage of bladder and kidney cancers has been identified. High hereditary predisposition for bladder and kidney cancer is presents in 4 and 5% respectively [2, 3]. Most cancers result from a complex interaction of environmental and genetic factors.

The genes *BRCA1* and *BRCA2* belongs to antioncogenes, inhibit the cell proliferation process, induce the process of apoptosis, maintain the stability of the genetic material, repair damage and changes that occur at the DNA level [4]. Mutations of *BRCA1* gene are the cause of 50–80% risk of breast, 40% ovarian cancer and in 40–50% of familial site-specific breast cancers [5–7]. Mutations in the *BRCA2* gene are responsible for tumors in breast cancer in 31–56%, ovarian cancer 11–27% and have been found in families with male breast cancers [8, 9]. Moreover changes in the *BRCA1* and *BRCA2* genes show an increased risk for the formation of neoplasms of other organs, including cancers of the: prostate, fallopian tube, pancreatic, bladder or melanoma [10–16].

To our knowledge, this is the first large-scale study to survey patients with bladder and kidney cancer for mutations in *BRCA1* gene (5328 insC, C61G and 4153 delA) and *BRCA2* (C5972T). To establish whether or not the mutations in the *BRCA1* and *BRCA2* genes contribute to bladder or kidney cancer in Poland, and to measure the impact of this variant on cancer risk, we genotyped 1028 unselected patients with bladder cancer, 688 unselected cases with renal cancer, 4000 healthy controls for testing the *BRCA1* gene and 2791 healthy controls for testing *BRCA2* gene.

## Material and methods

### Patients

We studied a series of 1028 unselected cases with urothelial bladder cancer (268 women and 760 men) and 688 unselected kidney cancer patients (284 women and 404 men) diagnosed at the Urology Hospital in Szczecin and the Genetic outpatients clinic between 2000 and 2018. A total of 1518 incident cases of bladder cancer and 869 kidney cancer were identified during the study period. Of these, 1419 patients with bladder (93%) and 835 with kidney cancer (96%) accepted the invitation to participate in the study. During the interview at the Genetic outpatients Clinic the goals of the study were explained, informed consent was obtained, family history and smoking status were collected, genetic counseling was given and a blood sample was taken for DNA analysis. The pathological diagnosis of bladder and kidney cancer was confirmed by biopsy review at a single central pathology laboratory in Szczecin, Poland. All cases were unselected for age, sex, smoking status and family history. The mean age of diagnosis for bladder cancer patients was 67 years (range 25–91) and was 64 years (range 17–91) for kidney cancer patients. A family history was taken by the construction a pedigree and questionnaire. A total of 40 patients with a family history of at least 1 bladder cancer in their first or second degree relatives and 27 cases with a family history of at least 1 kidney cancer in first or second degree relatives were identified. The vital status and the date of death of all of the cases were obtained from the Polish Ministry of the Interior and Administration in February 2021. In total we received information of 580 (56%) patients with bladder cancer and 233 (34%) with kidney cancer had died. All patients and control subjects are of European ancestry and are ethnic Poles. The study was approved by the Ethics Committee of Pomeranian Medical University in Szczecin.

### Controls

We used two control groups. The first control group included 4000 unselected, cancer-free individuals. These controls were selected to investigate the potential association between three Polish founder mutations of *BRCA1* gene (5328 insC, C61G and 4153 delA) and bladder and kidney cancer. This controls were taken from 1000 adult patient (age range 15–91, mean 58.3) lists of three family doctors from Szczecin, 1000 adults (age range 18–35, mean 24.3) from Szczecin who submitted blood for paternity testing and 2000 neonates from ten hospitals throughout Poland. This control group was described in detail elsewhere [17].

The second control group consisted of 2791 unselected, cancer-free individuals to estimate the association between the C5972T variant of *BRCA2* gene and bladder and kidney cancer. This control group consisted of 1993 newborn children from 10 hospitals throughout Poland and 798 adults (age range 19–89, mean 58.0) from Szczecin region unselected for family history. This control group was described in detail elsewhere [18].

To ensure comparability of the control groups, the allele frequencies was computed separately for the adult and neonatal control groups, and compared. The allele frequencies for *BRCA1* and *BRCA2* gene in our control groups were not dependent on age or sex, and the prevalence estimates of mutations in all genes were similar in younger and in older controls. Cases and controls are all residents of Szczecin and were Polish.

## Methods

DNA was extracted from peripheral blood for all cases and controls. The two mutations in *BRCA1* gene (5328 insC and 4153 delA) were detected by ASO-PCR analyses as described previously [19]. The third mutation in BRCA1 (C61G) and variant C5972T of *BRCA2* were genotyped with a TaqMan assay (Life Technologies, Carlsbad, CA) using a LightCycler Real-Time PCR 480 System (Roche Life Science, Mannheim, Germany). The mutations were confirmed by Sanger direct sequencing, using a BigDye Terminator v3.1 Cycle Sequencing Kit (Life Technologies), according to the manufacturer’s protocol. In all reaction sets, positive and negative controls (without DNA) were used.

## Statistical analysis

### Odds ratios

The prevalence of *BRCA1* and *BRCA2* allele were compared in bladder cancer cases and in controls, singly and in combination. Odds ratios were generated from two-by-two tables and statistical significance was assessed using the Fisher exact test where appropriate.

### Ethical statement

The study was performed in accordance with the principles of the Declaration of Helsinki. All patients and controls provided written informed consent.

## Results

### Bladder cancer

#### Gene *BRCA1*

The 1028 bladder cancer cases and 4000 controls were successfully genotyped for the *BRCA1* (5328 insC, C61G, 4153 delA) mutations (Table 1). The five (0.5%) carried a *BRCA1* mutation (all variants combined) (OR = 1.1; 95% CI 0.42–3.11; *p* = 1.0), including three (0.3%) cases with the 5328 insC mutation (OR = 0.8; 95% CI, 0.23–2.90; *p* = 0.8) and two (0.2%) patients with the C61G mutation (OR = 3.9; 95% CI, 0.54–27.7; *p* = 0.4). We did not find the mutation 4153 delA in any patient with bladder cancer.

**Table 1:** Effect of *BRCA1* and *BRCA2* mutations on bladder cancer risk

Mutation subjects	Number of carriers/total (frequency %)	OR	95% CI	***p***-value
***BRCA1***				
5328 insC				
Controls	14/4000 (0.003)	1.0		
Cases	3/1028 (0.003)	0.8	0.23–2.90	0.8
C61G				
Controls	2/4000 (0.0005)	1.0		
Cases	2/1028 (0.002)	3.9	0.54–27.7	0.4
4153 delA				
Controls	1/4000 (0.0002)	1.0		
Cases	0/1028	–	–	–
**All** ***BRCA1***				
Controls	17/4000 (0.004)	1.0		
Cases	5/1028 (0.005)	1.1	0.42–3.11	1.0
***BRCA2***				
C5972T				
Controls	159/2791(0.06)	1.0		
Cases	54/1028 (0.05)	0.9	0.66–1.26	0.6

The mutation 5328 insC was seen only in the group of 760 affected men (0.4%). The examined change was not observed in 40 family cases with bladder cancer in first- and/or second-degree relatives. Two patients with cancer cases and 5328 insC mutation died within a year of diagnosis and third to February 2021 was still alive.

#### Gene *BRCA2*

Of the bladder cancer patients enrolled in the study, fifty four (5.2%) carried a *BRCA2* mutation (C5972T) (OR = 0.9; 95% CI 0.66–1.26; *p* = 0.6) A C5972T mutation was seen in the 36 affected men (4.7%) and in 18 women (6.7%). Among cases with mutations 35 persons were smokers (5.2%). The mutation C5972T was seen in two family cases with bladder cancer in first- and/or second-degree relatives.

### Kidney cancer

#### Gene *BRCA1*

In total, 688 kidney cancer cases were genotyped for *BRCA1* mutations (5328 insC, C61G, 4153 delA) (Table 2). The three (0.4%) cases carried a *BRCA1* mutation (5328 insC) (OR = 1.2; 95% CI, 0.35–4.35; *p* = 1.0). The mutations C61G and 4153 delA had not been found among renal cancer patients.

**Table 2:** Effect of *BRCA1* and *BRCA2* mutations on kidney cancer risk

Mutation subjects	Number of carriers/total (frequency %)	OR	95% CI	***p***-value
***BRCA1***				
5328 insC				
Controls	14/4000 (0.003)	1.0		
Cases	3/688 (0.004)	1.2	0.35–4.35	1.0
C61G				
Controls	2/4000 (0.0005)	1.0		
Cases	0/688	–	–	–
4153 delA				
Controls	1/4000 (0.0002)	1.0		
Cases	0/688	–	–	–
**All** ***BRCA1***				
Controls	17/4000 (0.004)	1.0		
Cases	3/688 (0.004)	1.0	0.29–3.51	1.0
***BRCA2***				
C5972T				
Controls	159/2791(0.06)	1.0		
Cases	50/688 (0.07)	1.3	0.93–1.80	0.1

One man (0.2%) and two females (0.7%) were carriers of the mutation 5328 insC.

This man smoked and died one year after kidney cancer was diagnosed unlike women who had not smoked and to February 2021 was still alive.

#### Gene *BRCA2*

The mutation C5972T was seen in the group of fifty (7%) patients with kidney cancer, 28 (10%) women and 22 (5.4%) men, (OR = 1.3; 95% CI, 0.93–1.80; *p* = 0.1). 17 cases were smokers (6.5%). The mutation in gene *BRCA2* has been observed in two family cases with kidney cancer in first- and/or second-degree relatives.

## Discussion

The results of our unselected cohort 1028 bladder, 688 kidney cancer cases and two groups of controls 4000 and 2791 revealed no statistical significant difference, indicating that three mutations of *BRCA1* gene (5328 insC, C61G, 4153 delA) and one mutation of gene *BRCA2* (C5972T) do not seem to play a role in bladder or kidney cancer development.

Inherited changes in genes are the starting point for the development of almost all cancers. This was demonstrated for the first time in a breast cancer model that constitutional genetic markers associated with an increased risk of cancer occur in more than 90% of patients [20, 21]. The above data do not mean that only genetic factors are involved in carcinogenesis. It is already known that even at such a high risk of cancer as e.g. 80% risk of developing breast/ovarian cancer in carriers of the *BRCA1* mutation also constitute a significant share among carcinogenic factors environmental factors that may have the character of chemical factors (carcinogens contained in tobacco smoke, alcohol, arsenic concentration), physical (ionizing radiation, UV), biological (oncogenic HPV, EBV viruses) also constitute a significant share among carcinogenic factors [22]. Constitutional genetic changes most often predispose to cancers that develop as a consequence of the existence of multi-gene or monogenic predisposition, which may be associated with a high or moderately increased risk of developing cancer [23]. The cause of cancer may be mutation/ polymorphism in protooncogenes, suppressor genes (anti-oncogenes), DNA damage repair genes (mutator genes), or in genes that metabolize carcinogens and co-carcinogens.

In the literature there are several studies of mutation in the *BRCA1* and *BRCA2* genes and association with different cancers. Lubiński et al. observed in Polish patients based on studies of subsequent breast/ovarian cancers risks up to 75 years of age, respectively, about 66% for breast cancer and 44% for ovarian cancer [24]. The risk depends on the type of mutation and the location in the gene. The risk of developing breast cancer is about 2 times higher in carriers of the 5382insC mutation compared to the risk in carriers4153delA. An additional factor influencing the level of risk is the cancer family history. Metcalfe et al. found that the risk of breast cancer increases by another 20% for each first-degree relative who develops breast cancer before the age of 50. While the occurrence of ovarian cancer in any first or second degree relative is associated with a 60% higher risk of ovarian cancer [25].

Place of residence also affects the level of risk. In a prospective cancer risk study, penetration differences depending on the place of residence were found. The probability of developing breast cancer up to the age of 70 for carriers of the *BRCA1* gene mutation from North America was estimated at 72% and for carriers from Poland at 49%, indicating the importance of environmental factors [26]. Characteristic of ovarian cancers in carriers of the *BRCA1* mutation is also an increased risk of fallopian tube and peritoneal cancers, estimated at about 10%. Most likely, the risk of other organ cancers in some types of *BRCA1* mutations is also increased, however, this brca1-carrying effect has not been definitively proven so far. As shown by studies of 200 Polish families with strong aggregation of breast/ovarian cancers, constitutional mutations of the *BRCA2* gene are rare in this group, with a frequency of about 4%. The most of the *BRCA2* mutations occurring in our population, most probably, slightly increases the risk of breast cancer, although studies performed in New Medical Technology Center have shown that in families with aggregation of breast cancer diagnosed before the age of 50 and stomach diagnosed in men before the age of 55, the frequency of the *BRCA2* gene mutation is at the level of 10–20% [27]. Mutations in the *BRCA2* gene, are associated with a significant, although more precisely undefined, risk of: ovary, prostate cancer and cancers of the gastrointestinal tract: stomach, colon, pancreas [28]. This is supported by the research carried out in New Medical Technology Center, in which mutations were detected with a frequency of about 30% in families without breast cancer, but with aggregation of ovarian cancer and cancer of the stomach, colon or pancreas among relatives I^0^ lub II^0^ [28]. The research conducted in Poznań shows that the frequency of mutations of the *BRCA2* gene is also increased in families with breast cancer in men and it amounts to about 15% in Poland [29]. In a recent study, Nassar et al. demonstrated that the *BRCA2* gene is significant associated with bladder cancer [15]. They detected pathogenic variants of the *BRCA1* gene in 2.3% and the *BRCA2* gene in 2.1% in patients with urothelial carcinoma. Another report on 98 patients with bladder cancer showed that mutations in DNA repair genes (*CHEK2*, *ERCC5*, *GEN1*, *MLH1*, *PALB2*, *RAD50*, *RAD51B* and *RECQL4*) are associated with an unfavorable cancer prognosis and 22% of patients had a mutation [30]. In the Sweis et al. study, we noticed that changes to the *BRCA1, BRCA2, ERCC2* and *ATM* genes occur in 25% of urothelial carcinoma patients [14].

There are several strengths of our study including the large number of patients and controls, and the sampling of incident cases, unselected for age or family history. There is no reason to believe that age, sex or smoking behavior are important confounders of the observed association.

Our study was not without limitations, including a lack of information on histological features and smoking behavior in all patients.

## Conclusion

In conclusion, this study reveals that mutations in *BRCA1* gene (5328 insC, C61G, 4153 delA) and mutation (C5972T) of gene *BRCA2* did not seem to play a major role in bladder or kidney cancer development. Our results indicate that testing mutations are unlikely to be relevant for the identification of individuals at risk of bladder or kidney cancer, at least in the Polish population.

## Data Availability

Our data contain potentially sensitive information therefore we have not included it with our manuscript. Those who would like to request access to data may contact Melissa Sidhu at the Research Ethics Board of Women’s College Hospital by calling (416) 351–3732 × 2723 or email ac.latipsohcw@uhdis.assilem. The Pomeranian University of Medicine Ethics Committee will grant access to all researchers who meet the criteria for access to confidential data.
